# CRISPR/Cas as a Potential Diagnosis Technique for COVID-19

**Published:** 2020

**Authors:** Mahintaj Dara, Mahdieh Talebzadeh

**Affiliations:** Department of Molecular Medicine, Faculty of Advanced Medical Science and Technology, Shiraz University of Medical Sciences, Shiraz, Iran

**Keywords:** Coronavirus, COVID-19, CRISPR/Cas, SARS-CoV-2

Coronaviruses are positive single stranded RNA viruses, and are members of Coronaviridae family. Coronaviruses localize in respiratory tract and are usually known as common cold viruses ^1,2^. Seven strains of coronavirus family can infect humans and can cause different signs ranging from cold with major symptoms such as fever and sore throat to upper and lower respiratory tract infections resulting in pneumonia, severe respiratory tract infection and even death. These seven strains include HCoV-229E, HCoV-OC43, SARS-Co- V, human coronavirus NL63, human coronavirus HKU1, MERS-CoV and SARS-CoV-2, known as 2019-nCoV or “novel corona virus 2019” ^3^.

At present, “severe acute respiratory syndrome coronavirus 2” or “coronavirus disease 2019” (COVI D-19) which is closely related to SARS has become a global health problem. The first-ever COVID-19 case was identified in December 2019 in Wuhan, China; however, since then the virus has spread rapidly across the world and has become a worldwide pandemic and an international concern ^4^.

To the best of our knowledge until March 2020, COVID-19 has been reported in 161 countries. COVID-19 is typically transmitted by respiratory droplets during sneezing and coughing ^5^. There is no evidence of vertical transmission or transmission during pregnancy ^6,7^. Incubation period of COVID-19 is estimated between 2–14 days and during this time, infected peoples are considered as asymptomatic carriers. Although infection may be asymptomatic, patients typically have fever, cough and shortness of breath. Occasionally disease progresses acutely and causes severe pneumonia, multiple organ failure and death ^8^. Patients with underlying medical conditions such as heart and respiratory diseases, asthma, diabetes and immunodeficiency diseases, in addition to elderly age group are high risk and more susceptible to COVID-19 infection ^9^. At present, there is no certain treatment or vaccination for prevention of COVID-19 and infected people are either isolated or, in critical conditions, take nonspecific or supportive care ^9^. Diagnosis is made based on the symptoms of the disease, chest CT (Computed tomography scan) scan and qRT-PCR (Quantitative reverse transcription polymerase chain reaction). qRT-PCR technique is the current COVID-19 (SARS-CoV-2) gold standard molecular detection method approved by CDC and World Health Organization (WHO) ^10,11^. Recently, researchers have proposed a coronavirus rapid detection method based on CRISPR/Cas system ^12^. CRISPR/Cas (Clustered Regularly Interspaced Short Palindromic Repeats) is an adaptive immune system in archaea and bacteria against foreign genetic elements such as phages. Recently, CRISPR/Cas has become a powerful gene editing tool and a promising treatment for genetic diseases and cancers ^13,14^. In this technique, a programmable protein attaches to the target site by a guide RNA for cleavage of the target sequence. There are several types of Cas proteins that have different properties. Among them, Cas9 protein has received more attention for gene editing whereas, Cas12a and Cas13a have been more efficient in diagnosis of diseases ^15,16^. Cas12a is DNA-specific but Cas13a works with RNA which makes it convenient in detection of SARS-CoV-2. Recently, Zhang *et al* reported specific high-sensitivity enzymatic reporter unlocking (SHERLOCK) technology which is a CRISPR/Cas13 based nucleic acid detection technique for rapid detection of SARS-CoV-2 ^17,18^.

They targeted S and ORF1ab protein genes in coronavirus genome. Cas13 identifies and binds to previously determined target sequence which leads to fairly random cleavage of surrounding ssRNA molecules. SHERLOCK technology utilizes a quenched fluorescent ssRNA reporter. The presence of ssRNA corona-virus genome in samples activates Cas13 resulting in the production of quantifiable signals. Amplification of targeted DNA or RNA by Recombinase Polymerase Amplification (RPA) or reverse transcriptase-RPA (RTRPA) prior to the start of reaction improves the sensitivity of the assay. Subsequently, amplified DNA is converted to RNA by combination of RPA and T7 transcription. Ultimately, detection is made by simultaneous incorporation of the ssRNA reporter (Biotin-RNA-FITC). Viral genome is detected at attomolar concentration using SHERLOCK technology ^19^. The test can be carried out starting with RNA purified from patient samples, as used for qRT-PCR assays, and can be read out using a dipstick in less than an hour, without requiring elaborate instrumentation ^18^. As a result, application of CRISPR/Cas13 based diagnosis or SHERLOCK for SARS-CoV-2 detection is much faster than detection by qRT-PCR and has high sensitivity. Consequently, SHERLOCK technology could swiftly replace qRT-PCR technique considering the high demand for rapid diagnosis tests in current global pandemic state of COVID-19.
